# Observed Trace Mineral Deficiencies in a Group of Locally Harvested Sheep in Hawai’i

**DOI:** 10.3390/vetsci12101002

**Published:** 2025-10-16

**Authors:** Shaye N. R. Nishimura, Janae S. Bulosan, Mark S. Thorne, Melelani A. Oshiro, Jenee S. Odani, Caleb C. Reichhardt

**Affiliations:** 1 Department of Human Nutrition, Food and Animal Sciences, University of Hawai’i at Mānoa, Ag Sciences Building 302A, 1955 East-West Road, Honolulu, HI 96822, USAthornem@hawaii.edu (M.S.T.);; 2 Animal and Veterinary Services Program, University of Hawai’i, Honolulu, HI 96822, USA

**Keywords:** micro-minerals, nutrition, ovine, deficiency, tropical, supplementation

## Abstract

**Simple Summary:**

There is a significant gap in the information available about the trace mineral status of sheep in tropical and subtropical environments. This research found that of the animals sampled at harvest, 31% were deficient in cobalt, 47% were deficient in copper, and 45% were deficient in iron. In the broader context, these findings highlight the need for region-specific mineral management strategies that account for environmental challenges such as parasite pressure, which can impair mineral absorption and contribute to widespread deficiencies, even when dietary supply appears adequate. For Hawai’i sheep producers, it is suggested that they be aware of deficiency signs for Co, Cu, and Fe, as they appear to be common in sampled sheep in the state.

**Abstract:**

Trace minerals (TM) play a critical role in the health and productivity of small ruminants. They are essential for various physiological functions, including growth, reproduction, and immune response, yet research on their status in Hawai’i is notably limited. This study focused on surveying the current trace mineral concentrations of locally raised and harvested sheep to identify common deficiencies and toxicities. Sheep liver (*n* = 83) and plasma (*n* = 79) samples were collected over eight months from local harvest facilities and private operations. There was a high percentage of liver samples that were deficient in copper (47%), iron (46%), and cobalt (31%). There was a low percentage of liver samples that were toxic in Mn (11%). Strong positive correlations in plasma zinc and plasma magnesium (*r* = 0.814, *p* < 0.0001) and liver molybdenum and liver selenium (*r* = 0.72, *p* < 0.0001) were found. With this data, local small ruminant producers will be able to evaluate their nutrition management program. Addressing these gaps is vital for conducting future research studies, improving sheep health, and ensuring the productivity of small ruminant operations in Hawai’i.

## 1. Introduction

In the U.S., the number of sheep operations is increasing, with the majority of these producers having less than 10 years of experience [1]. Similarly, there is a growing number of small ruminant operations in Hawai’i [2]. In a national survey in the U.S., it was found that breeding stock and lamb nutrition are extremely important for producers [1]. However, there has been a lack of published research trials looking at small ruminant production regarding the trace mineral status of sheep in tropical and subtropical environments. Globally, mineral deficiencies are known to impact ruminants. In the Campos Gerais region of Brazil, 26% of sampled dairy cows were found to be deficient in cobalt [3]. In Northwest Iran, 87% of sheep and goats sampled were deficient in Selenium [4]. Additionally, goats raised around lead–zinc smelters have been found to have lowered blood copper and cobalt concentrations [5]. More locally, cattle raised in Hawai’i tend to be deficient in copper [6]. In Hawai’i, unlike cattle, sheep have not received the same level of attention in terms of research on nutrition. This lack of information challenges local sheep producers who rely on trial and error, community knowledge, and other external resources that may not accurately reflect conditions.

Understanding the nutritional needs of sheep is essential for maintaining flock health and productivity. Trace minerals play a critical role in meeting these needs, supporting key physiological functions that promote normal growth and development. However, interactions among trace minerals can significantly influence their bioavailability, with some minerals competing for absorption and potentially inhibiting the uptake of others. Other external factors, such as mineral supplementation [7], forage and soil mineral composition, and climate [8,9], also affect the mineral concentrations in sheep. Assessing the trace mineral status is vital for identifying imbalances, allowing producers to prevent and minimize nutritional issues that could impact the health and performance of their flock. Therefore, the primary goal of this research was to identify common mineral deficiencies and toxicities in harvested Hawai’i sheep in liver and plasma samples collected following harvest. The liver is primarily a storage and regulatory organ, and it tends to accumulate higher concentrations of minerals such as Fe, Cu, and Zn [10]. On the other hand, plasma serves as a transport medium for minerals, and its values are more tightly regulated [11]. Understanding common mineral deficiencies and toxicities observed will allow for future management goals and decisions to be developed to address these nutritional imbalances to assist with further improving small ruminant production in the state.

## 2. Materials and Methods

### 2.1. Farm Profile

From September 2023 to April 2024, liver and plasma samples from 83 individual sheep (N = 83) were collected from 11 different farms across two of the eight Hawai’ian Islands, O’ahu and Hawai’i Island, following harvest (Table 1). Due to a sampling issue with 4 sheep, only livers were collected; as such, this study analyzed 83 sheep livers (*n* = 83) and 79 plasma samples (*n* = 79) for common mineral deficiencies. Compared to the number of sheep harvested in 2021, these 83 samples accounted for 6.4% of the animals harvested at a USDA-inspected facility [12]. The samples were obtained from two local processing facilities and one private operation. Animals were intended for human consumption and did not die from disease or other conditions. Accessibility and availability of the samples determined the different number of samples collected. Therefore, samples were collected at random and not chosen from specific farms.

The sheep sampled in this study represented early fall to late winter mineral status. The samples collected were from male and female adults and lambs raised for meat and were harvest-ready. The number of samples per farm ranged from one to 18, with 10 out of the 11 farms collecting the same number of both liver and plasma samples (Table 1). However, while nine liver samples were collected from Farm G, only five plasma samples were collected due to difficulties with the collection of four individual sheep.

With how the samples were collected (following slaughter), and as we were primarily working with slaughterhouses, we were unable to collect forage and nutritional data on the sheep analyzed. Producers were asked directly or through email whether they gave mineral supplements to their flock. Four out of the 11 farms that were sampled stated that they gave mineral supplements to their flock. The other seven farms did not respond to specify whether they supplemented minerals or not. The producers were not asked to specify the type, brand, amount, or frequency they gave to their sheep due to privacy reasons.

### 2.2. Sample Collection

For each animal, one liver sample, approximately five grams from the left lobe, and one blood sample, 6 mL, were collected postmortem. The liver samples were placed in Whirl-Pak bags and placed in a cooler immediately after collection. Upon arrival at the laboratory, the liver samples were stored at −80 °C for long-term storage and analysis. Blood was collected during exsanguination using 6 mL, 13 × 100 mm BD Vacutainer plasma blood collection tubes containing trace mineral grade K_2_EDTA (Franklin Lakes, NJ). Blood samples were centrifuged at 1000× *g* for 15 min at 4 °C. Plasma was then collected, aliquoted, and stored at −80 °C until further analysis. Each plasma tube and liver bag was labeled with the collection date, animal identification number, and farm code.

### 2.3. Mineral Analysis

Following the collection of liver and plasma, samples were sent for analysis at Washington Animal Disease Diagnostic Laboratory (Pullman, WA, USA) using inductively coupled plasma mass spectrometry (ICP-MS). The liver samples were analyzed through a tissue mineral screen using nitric acid digest and analyzed using an Agilent ICP-MS 7800 with Agilent SPS-4 auto sampler. The Argon flow was 15 L/min, the nebulizer flow was 1.09 L/min, the plasma power was 1550 W, the helium flow was 2 mL/min, and the hydrogen flow was 4.5 mL/min. Reference screens for the liver were freeze-dried liver with a known reference range. Liver elements were selected based on the packages that the veterinary diagnostic lab offered. The liver elements screening panel included the following 11 trace minerals: arsenic (As), barium (Ba), cadmium (Cd), chromium (Cr), cobalt (Co), copper (Cu), iron (Fe), manganese (Mn), molybdenum (Mo), selenium (Se), and zinc (Zn).

Plasma samples were analyzed through a serum mineral screen using protein precipitation. Plasma samples were analyzed on a Perkin Elmer ICP-Optical Emission Spectrometry (OES) 8300 with Cetac ASX-520 autosampler. The Argon flow was 15 L/min, the nebulizer flow was 0.73 L/min, the plasma power was 1500 W, with an axial plasma view, and a sample flow rate of 1.5 mL/min. Plasma minerals were selected based on the mineral packages that the veterinary diagnostic lab offered. The plasma mineral screening panel observed the following three trace minerals: Cu, Fe, and Zn. Macro-minerals such as calcium (Ca), magnesium (Mg), and phosphorus (P) were also observed in the plasma mineral screening panel. Quality control followed a process whereby, for every 10 samples run, one sample duplicate preparation was run, along with running one matrix-matched reference material. The matrix-matched reference was internally developed and validated alongside reference materials from Seronorm (SERO AS, Hvalstad, Norway).

### 2.4. Statistical Analysis

Descriptive analysis of liver and plasma trace mineral concentrations was conducted using the PROC UNIVARIATE procedure (Table 2 and Table 3). Normality was evaluated using the Shapiro-Wilk test. Pearson correlation coefficients between the liver and plasma concentrations were calculated using the PROC CORR procedure. Descriptive and correlation analyses were conducted using SAS software (version 9.4; SAS Institute Inc., Cary, NC, USA). Statistical significance was declared at *p* < 0.05, and the tendencies were noted at 0.05 < *p* ≤ 0.10. The percentage of samples in the adequate, deficient, and toxic ranges was calculated using Microsoft Excel (Version 16.95.1), relevant to known concentrations [11].

## 3. Result

### 3.1. Descriptive Analysis of Plasma and Liver Concentrations

The trace minerals Ba, Cd, Cr, Co, Cu, Fe, Mn, Mo, Se, and Zn were evaluated in the liver panel (Table 2). The mean concentration for Cu was 49.730 µg/g with a standard deviation (SD) of 28.704 µg/g, while the mean for Fe was 66.346 µg/g (SD = 28.868 µg/g), both having the highest concentrations among the trace minerals. In contrast, the mean for Zn was 38.718 µg/g (SD = 6.750 µg/g). The mean values for the other trace minerals were 3.181 µg/g (SD = 0.864 µg/g) for Mn, 0.819 µg/g (SD = 0.446 µg/g) for Mo, 0.465 µg/g (SD = 0.159 µg/g) for Se, and 0.040 µg/g (SD = 0.024 µg/g) for Co. Additionally, the mean concentrations of Ba, Cd, and Cr were 0.042 µg/g (SD = 0.021 µg/g), 0.049 µg/g (SD = 0.027 µg/g), and 0.040 µg/g (SD = 0.040 µg/g).

The trace minerals Cu, Fe, and Zn, along with macro-minerals Ca, Mg, and P, were evaluated in the plasma panel (Table 3). The mean concentration of Cu was 0.811 µg/g with an SD of 0.195 µg/g, while the mean for Fe was 1.138 µg/g (SD = 0.415). The mean concentration of Zn was 1.361 µg/g. The mean concentrations of Ca were the highest overall, at 86.764 µg/g with an SD of 9.082 µg/g. Phosphorus had a mean of 64.623 µg/g (SD = 16.014 µg/g). Magnesium had a mean of 22.55 µg/g (SD = 4.585 µg/g).

### 3.2. Adequate, Deficient, and Toxic Concentration Ranges

**Figure 1:**
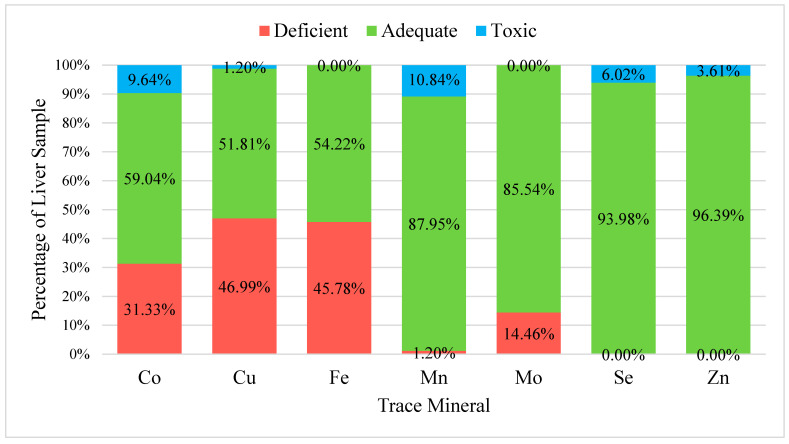
Sheep Liver Sample Percentages in the Adequate, Deficient, and Toxic Trace Mineral Concentration Range. Analysis of sheep liver sample (*n* = 83) percentages in the adequate, deficient, and toxic trace element concentration range for seven trace elements: cobalt (Co; adequate range: 0.08–0.35 µg/g, copper (Cu; adequate range: 200–600 µg/g), iron (Fe; adequate range: 250–1000 µg/g), manganese (Mn; adequate range: 3.5–20 µg/g), molybdenum (Mo; adequate range: 0.9–7.5 µg/g), selenium (Se; adequate range: 0.8–3.0 µg/g), and zinc (Zn; adequate range: 80–300 µg/g). Values are expressed as percentages on a dry tissue basis. Reference ranges were adapted from [11] for ovine hepatic trace mineral concentrations. Samples were taken from locally harvested sheep from local harvest facilities in Hawai’i.

Reference ranges for sheep liver and plasma trace mineral concentrations were taken from T. Herdt and B. Hoff [11]. In the 83 liver samples, the trace minerals evaluated were Co, Cu, Fe, Mn, Mo, Se, and Zn (Figure 1). Five out of the seven trace minerals that were analyzed were in the deficient range. Copper was the mineral found to be the most deficient in 47% of the liver samples, followed by Fe (45.78%), Co (31.3%), Mo (14.5%), and Mn (1.2%). Selenium and Zn did not have any samples that were deficient. Zinc had the highest percentage of adequate samples (96.39%), followed by Se (93.98%), Mn (87.95%), Mo (85.54%), Co (59.04%), Fe (54.22%), and Cu (51.81%). Five out of the seven trace minerals that were analyzed fell within the toxic range. Manganese was the mineral found to be the most toxic in 10.8% of the liver samples, followed by Co (9.6%), Se (6.02%), Zn (3.6%), and Cu (1.2%). Overall, the trace minerals that had a large percentage of deficient samples were Cu, Fe, and Co, while the trace minerals that had a large percentage of adequate samples were Zn and Se.

**Figure 2:**
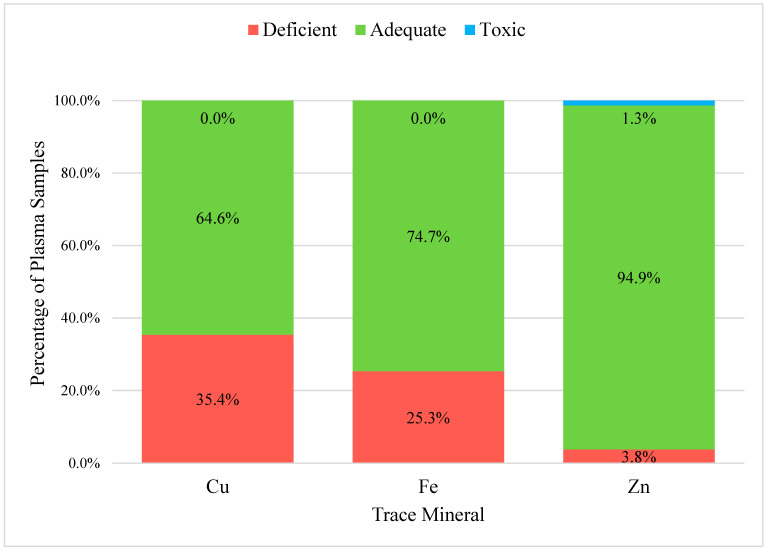
Sheep Plasma Sample Percentages in the Adequate, Deficient, and Toxic Trace Mineral Concentration Range: Analysis of sheep plasma samples (*n* = 79) percentages in the adequate, deficient, and toxic trace mineral concentration range for three trace minerals: copper (Cu), iron (Fe), and zinc (Zn). Reference ranges were adapted from [11]. For Cu, adequate was considered between 0.75 and 1.7 µg/mL, for Fe, adequate was considered between 0.9 and 2.7 µg/mL, and for zinc, adequate was determined to be between 0.55 and 1.2 µg/mL. Samples were taken from locally-harvested sheep from local harvest facilities in Hawai’i.

In the 79 plasma samples, the trace minerals evaluated were Cu, Fe, and Zn (Figure 2). The percent adequate, deficient, and toxic was not calculated for the macro-minerals. Ca, Mg, and P. All three trace minerals had high average percentages of adequate samples. Zinc was found to be adequate in 94.9% of the plasma samples, followed by Fe (74.7%) and Cu (64.6%). All three trace minerals had a small percentage of deficient samples. Copper was the mineral found to be deficient in 35.4% of the plasma samples, followed by Fe (25.3%) and Zn (3.8%). Only a very small percentage of samples were in the toxic range for Zn (1.3%). There were no other trace minerals that were in the toxic range.

Each of the 11 individual farms had a varied number of samples, as well as concentration ranges of both liver and plasma samples that were adequate, toxic, and deficient. Thirty-three percent of Farm A’s (*n* = 3) liver and plasma samples were deficient in Cu, and 33% of liver samples were toxic in Se. Farm B (*n* = 9) had 56% of its liver samples and 22% of its plasma samples deficient in Cu, 33% of liver samples deficient in Mo, while 11% of the Co, Mn, and Zn concentrations of the liver samples each were toxic. While 100% of Farm B liver samples were adequate in Fe, 11% of its plasma samples were deficient in this mineral. Farm C (*n* = 18) had 72% of its liver samples that were deficient in Co, 50% of liver samples and 11% of plasma samples deficient in Cu, 39% of liver samples deficient in Fe, 44% of liver samples deficient in Mo, and 39% of liver samples toxic in Mn. Farm D (*n* = 2) had 50% of its liver samples deficient in Co and Fe, and 50% of its liver samples toxic in Se. Farm E (*n* = 14) had 21% of its liver samples deficient in Co and 78% of samples deficient in Fe. All of Farm G’s (*n* = 9) liver samples were deficient in Cu, 78% deficient in Fe, 78% toxic in Co, 11% toxic in Mn, 11% toxic in Se, and 22% toxic in Zn. Farm H (*n* = 6) had 33% of its liver samples and 17% of plasma samples deficient in Cu, 50% of liver samples and 17% of plasma samples deficient in Fe, and 17% of liver samples toxic in Se. Fifty percent of Farm I’s (*n* = 10) liver samples were deficient in Co, Cu (90%), Fe (10%), Mn (10%), and Mo (10%). Farm J (*n* = 8) had 13% of its liver samples deficient in Co, 38% deficient in Cu, 62% deficient in Fe, and 12% toxic in Se, while 12% of plasma samples were deficient in Fe. All of Farm K’s (*n* = 3) liver samples were deficient in Co and Fe, while 33% were toxic in Cu.

### 3.3. Liver and Plasma Correlations

All liver and plasma trace mineral concentration samples were analyzed for Pearson correlation coefficient involving liver–liver (Table 4), liver–plasma (Table 5), and plasma–plasma correlations (Table 6). Plasma Zn and plasma Mg had the strongest positive correlation (*r* = 0.814; *p* < 0.0001). Liver Se and liver Mo had the second strongest positive correlation (*r* = 0.720; *p* < 0.0001). Plasma Fe and liver Fe had a moderate positive correlation (*r* = 0.427; *p* = 0.0002). Plasma Cu and liver Cu did not have a significant correlation (*r* = 0.120; *p* = 0.306). Plasma Zn and liver Zn also did not have a significant correlation (*r* = −0.023; *p* = 0.844). Plasma Zn and plasma P had the strongest negative correlation (*r* = −0.462; *p* < 0.0001), plasma P and plasma Mg had the second strongest negative correlation (*r* = −0.389; *p* = 0.0005).

## 4. Discussion

The objective of this research was to identify common trace mineral deficiencies and toxicities to address the significant gap in the information regarding the trace mineral status of sheep in Hawai’i. Understanding the specific nutritional needs of sheep, particularly with the rise of small ruminant livestock operations, is crucial for ensuring their health and growth. This research found that 31% of the sheep liver analyzed were deficient in cobalt, 47% were deficient in copper, and 46% deficient in iron. The results will allow local sheep producers to use this information to watch for these specific deficiency signs associated with these mineral imbalances, and address them if they believe their flocks may be impacted by them.

Cobalt deficiency is one of the more common mineral deficiencies observed in ruminant livestock grazing in a tropical environment [13]. In the current trial, 31% of the livers collected from harvested sheep were deficient in cobalt. Cobalt’s primary function is vitamin B12 production, which has a variety of functions, including metabolism of propionate, amino acids, and one-carbon metabolism. Animals that are deficient in cobalt typically have decreased growth, intake, and efficiency [14], but symptoms can take years to present themselves. For grazing lambs, it has been found that their dietary cobalt is typically not met from pasture alone [15]. Research has shown that drenching ewes with cobalt every two weeks improves cobalt vitamin B12 status better than a single bolus [16]. Additionally, supplementation of Co to grazing lambs improved lamb performance after weaning [17], highlighting the benefit of supplementing Co to grazing sheep.

Iron is a key component of hemoglobin and enzymes involved in oxygen transport and electron transfer [18]. However, the large SD observed for Fe suggests that its concentrations can vary widely across individual sheep, most likely reflecting differences in dietary intake or gastrointestinal nematode infection. This variability could also be influenced by underlying conditions such as anemia or hemochromatosis, which are known to impact Fe metabolism [19]. Despite the high Fe concentrations in Hawai’i’s forages [6], 45% of the samples that were in the Fe-deficient range from this study suggest there may be poor absorption of Fe, possibly due to parasitic infections. Parasite infections are known to impact hemoglobin concentrations in sheep [20]. Hawai’ian sheep are very susceptible to gastrointestinal nematodes due to favorable year-round weather conditions [21]. Therefore, parasitic infections are very hard to overcome and are a constant challenge for Hawai’i producers. In lambs infected with *Haemonchus contortus*, lambs receiving intramuscular dosages of iron dextrose had increased iron stores and reduced severity of anemia that was associated with the parasite infection [22]. This suggests that iron supplementation may be one possible method to improve both iron status and parasite load of Hawai’i sheep.

Of the samples analyzed, there was a large SD for Cu, which demonstrates the significant variability in its concentration. This variability could be due to differences in Cu absorption, liver function, or other factors such as genetic variation [19]. However, 47% of all liver samples analyzed and 35% of all plasma samples analyzed were deficient in Cu. One possible explanation for this is the low Cu availability in Hawai’i’s forages [6]. Similarly to iron, gastrointestinal parasites impact serum Cu concentrations in sheep [20]. Therefore, improved parasite management practices may be beneficial to improve the mineral status of sheep in Hawai’i. It is important to note that since these samples were collected from harvest, the gastrointestinal parasite load was unknown. Copper oxide wire particles have been found to be an effective method of parasite management for sheep [23]. Copper oxide wire particles have been shown to increase serum concentrations of copper in sheep and to be a safe treatment for improving the copper status of sheep without sheep experiencing toxic effects of Cu [24].

It is of interest to highlight that the three most commonly deficient minerals in the sampled population, Co, Cu, and Fe, are known to interact with each other. In sheep receiving low Co diets, their muscle has decreased Cu and Fe content, highlighting the fact that Co is important absorption and accumulation of other minerals [25]. Similarly, short-term Cu supplementation increases Fe absorption in sheep [26], and in Merino rams, seminal plasma Cu and seminal plasma Fe have been found to be correlated [27]. This suggests that low Cu and Co may be influencing the Fe status of the sheep, as Hawai’i forages are known to be high in Fe concentrations [6].

Discussing the interplay between minerals, the strongest positive correlation observed in this study was between plasma Zn and plasma Mg. The strong relationship between Zn and Mg could be influenced by their shared dietary sources. A study done by Quillian et al. [28] found that an increase in dietary Mg intake affects Zn metabolism, which leads to changes in Zn retention in Holstein calves. For the sheep in this study, the strong correlation between plasma Zn and Mg suggests that dietary intake or metabolic regulation of Mg may influence the status of Zn, indicating the need to consider these interactions when evaluating mineral adequacy and designing supplementation programs.

While deficiencies were a concern, there was a low percentage of samples that were in the toxic concentration range found in the liver. Manganese had the highest percentage of toxic samples, followed by Co and Se. Diets containing concentrations of more than 2000 ppm of may cause Mn poisoning [18]. Additionally, the consumption of organic Mn can cause an increase in liver concentration in ewes and lambs [29,30]. These findings may indicate that some individuals are exposed to excess concentrations of certain trace minerals. Although Mn is one of the least toxic minerals [31], the deficiency in Fe may be causing Mn toxicity [11], possibly due to the antagonistic relationship between the two minerals [32].

A major limitation of this study is that only four out of the 11 farms gave a clear indication of feeding their sheep mineral supplements. Factors such as soil mineral concentration, forage mineral concentration, climate, and supplementation practices all influence the mineral status of sheep [33]. Although the specific method, brand, amount given, or frequency of supplementation was not mentioned, it is likely that the mineral supplements used had an impact on the mineral concentrations observed. As such, the effect of mineral supplementation was not evaluated in the present study. Research has found that in tropical regions, grazing hair sheep not supplemented with trace minerals are deficient in them [34]. Of note, all farms had animals that were deficient in at least one element analyzed. Forage mineral compositions will also influence the concentrations of trace minerals observed in sheep [33]. With over 700,000 acres of land in the state of Hawai’i dedicated to grazing [35], not all areas will have the same mineral profiles due to the islands’ varied environments. This includes the different types of forages found across the islands, such as kikuyu grass, pangola grass, guinea grass, California grass, and much more [35]. Minerals in the soil will also influence the forage mineral composition, which will ultimately influence the mineral status of sheep. Hawai’i has 10 of the 12 soil orders in the world [36]. Andisols, the dominant soil type in Hawai’i, are high in organic matter and have great water retention capabilities, making them well-suited for supporting the growth of forages in the state. All in all, recognizing the impact of Hawai’i’s varied environments on mineral availability is crucial to improving the management and well-being of sheep in the state. As such, producers should analyze both their forages and animals, and design a mineral management program that maximizes and meets the goals of their operations. Future studies are needed to take into account the effect of supplement strategy, soil mineral content, and forage mineral content on their resulting impact on Hawai’i sheep’s mineral status. However, there is limited information available focused on the trace mineral status of sheep in Hawai’i’s tropical and sub-tropical environment. The 83 samples collected from the account for 6.4% of the animals harvested at a USDA-inspected facility, compared to the number of sheep harvested in 2021 [12]. This research provides a baseline for producers to use to evaluate mineral deficiencies in their flocks, and common mineral deficiencies observed in Hawai’ian sheep (cobalt, copper, and iron).

## 5. Conclusions

The objective of this study was to identify common deficiencies and toxicities of trace minerals in Hawai’i sheep. This study provides valuable information on the trace mineral status of sheep in Hawai’i. This research found that of the animals sampled at harvest, 31% were deficient in cobalt, 47% were deficient in copper, and 45% were deficient in iron. The farm-specific variations in mineral concentrations highlight the need for customized nutrition management strategies that consider the unique environment and dietary factors on each farm. In the broader context, these findings highlight the need for region-specific mineral management strategies that account for environmental challenges such as parasite pressure, which can impair mineral absorption and contribute to widespread deficiencies, even when dietary supply appears adequate. Given the significant imbalances observed, this study emphasizes the importance of continued research into the trace mineral requirements of small ruminants in Hawai’i, as well as the need for tailored feeding practices to improve small ruminant health, productivity, and the overall sustainability of small ruminant operations. For Hawai’i sheep producers, it is suggested that they be aware of deficiency signs for Co, Cu, and Fe, as they appear to be common in sampled sheep in the state. Additional research to identify supplementation strategies for these deficient minerals will be beneficial for local Hawai’ian producers.

## Figures and Tables

**Table 1:** The different farms with the number of samples from each farm and whether they self-reported providing mineral supplements to their flock.

Farms	Number of Samples	Provided Mineral Supplements
A	3	No Response
B	9	No Response
C	18	Yes
D	2	No Response
E	14	Yes
F	1	No Response
G	9	Yes
H	6	No Response
I	10	No Response
J	8	Yes
K	3	No Response

**Table 2:** Descriptive statistics for trace mineral concentrations in harvested sheep livers (*n* = 83) in Hawai’i.

Liver Descriptive Statistics					
	Mean	SD	Sum	Min	Max
Ba (µg/g)	0.042	0.021	3.345	0.010	0.130
Cd (µg/g)	0.049	0.027	3.822	0.010	0.110
Cr (µg/g)	0.040	0.020	3.266	0.018	0.150
Co (µg/g)	0.040	0.024	2.844	0.011	0.100
Cu (µg/g)	49.730	28.704	3929	2.900	120.000
Fe (µg/g)	66.346	28.868	5175	27.000	140.000
Mn (µg/g)	3.181	0.864	241.820	0.520	5.700
Mo (µg/g)	0.819	0.446	67.143	0.027	1.800
Se (µg/g)	0.465	0.159	36.740	0.210	0.760
Zn (µg/g)	38.718	6.750	3020	27.000	72.000

**Table 3:** Descriptive statistics for trace mineral concentrations in harvested sheep plasma (*n* = 79) in Hawai’i.

Plasma Descriptive Statistics					
	Mean	SD	Sum	Min	Max
Ca (µg/g)	86.764	9.082	6768	47.500	104.000
Cu (µg/g)	0.811	0.195	62.440	0.370	1.300
Fe (µg/g)	1.138	0.415	86.450	0.230	2.100
Mg (µg/g)	22.551	4.585	1714	15.000	53.900
P (µg/g)	64.623	16.014	4976	1.000	97.100
Zn (µg/g)	1.361	4.464	104.820	0.490	40.000

**Table 4:** Estimated Pearson correlation coefficient and *p*-value of liver trace element concentrations in samples from Hawai’ian sheep.

Liver Trace Elements											
		Ba	Cd	Cr	Co	Cu	Fe	Mn	Mo	Se	Zn
**Liver Trace Elements**	**Ba**	1.00 ^1^ 0 ^2^	0.208 0.073	−0.039 0.748	−0.163 0.184	0.042 0.721	0.066 0.572	0.040 0.735	−0.185 0.103	−0.233 **0.042**	0.159 0.172
	**Cd**	-	1.00 0	−0.070 0.544	−0.257 **0.036**	0.078 0.508	−0.050 0.673	0.051 0.670	−0.033 0.773	−0.199 0.087	0.163 0.166
	**Cr**	-	-	1.00 0	0.061 0.617	−0.345 **0.002**	0.072 0.533	0.143 0.329	−0.070 0.538	−0.135 0.237	0.074 0.523
	**Co**	-	-	-	1.00 0	−0.368 **0.002**	0.179 0.145	−0.168 0.167	0.298 **0.012**	0.463 **<0.0001**	0.199 0.330
	**Cu**	-	-	-	-	1.00 0	−0.214 0.065	−0.119 0.314	0.170 0.135	0.086 0.463	−0.141 0.226
	**Fe**	-	-	-	-	-	1.00 0	−0.058 0.624	−0.243 **0.032**	−0.093 0.430	0.235 **0.042**
	**Mn**	-	-	-	-	-	-	1.00 0	−0.362 **0.001**	−0.370 **0.001**	−0.180 0.125
	**Mo**	-	-	-	-	-	-	-	1.00 0	0.720 **<0.0001**	−0.015 0.896
	**Se**	-	-	-	-	-	-	-	-	1.00 0	0.022 0.849
	**Zn**	-	-	-	-	-	-	-	-	-	1.00 0

^1^ The top number indicates the coefficient (*r*). ^2^ The bottom number indicates the significance (*p*-value).

**Table 5:** Estimated Pearson correlation coefficient and *p*-value of liver and plasma trace element concentrations in samples from Hawai’ian sheep.

Plasma Trace Elements							
		Ca	Cu	Fe	Mg	P	Zn
**Liver Trace Elements**	**Ba**	0.041 ^1^ 0.726 ^2^	−0.091 0.440	0.175 0.135	−0.014 0.901	−0.017 0.887	−0.0006 0.996
	**Cd**	−0.161 0.157	0.074 0.535	−0.011 0.923	−0.131 0.271	0.208 0.078	−0.132 0.266
	**Cr**	−0.121 0.294	−0.070 0.554	0.023 0.842	−0.150 0.197	0.104 0.372	−0.064 0.582
	**Co**	−0.004 0.974	0.082 0.509	0.139 0.264	−0.071 0.573	−0.118 0.340	0.056 0.654
	**Cu**	0.120 0.306	0.216 0.064	−0.214 0.069	0.042 0.733	0.153 0.189	−0.130 0.268
	**Fe**	−0.078 0.507	0.057 0.629	0.427 **0.0002**	0.138 0.274	−0.385 **0.0008**	0.252 **0.031**
	**Mn**	0.155 0.195	−0.326 **0.006**	0.303 **0.011**	−0.091 0.452	0.064 0.597	−0.118 0.326
	**Mo**	0.122 0.289	0.294 **0.009**	−0.171 0.140	0.092 0.430	0.152 0.319	0.125 0.280
	**Se**	0.296 **0.009**	0.036 **0.002**	−0.168 0.152	0.0121 0.919	0.056 0.633	0.003 0.983
	**Zn**	−0.180 0.125	0.359 **0.001**	−0.225 0.057	−0.221 0.062	−0.156 0.188	−0.023 0.844

^1^ The top number indicates the coefficient (*r*). ^2^ The bottom number indicates the significance (*p*-value).

**Table 6:** Estimated Pearson correlation coefficient and *p*-value of plasma trace element concentrations in samples from Hawai’ian sheep.

Plasma Trace Elements							
		Ca	Cu	Fe	Mg	P	Zn
**Plasma Trace Elements**	**Ca**	1.00 ^1^ 0 ^2^	0.235 **0.040**	0.001 0.990	0.0009 0.994	0.317 **0.005**	−0.007 0.949
	**Cu**	-	1.00 0	−0.318 **0.005**	0.087 0.457	0.030 0.794	0.161 0.164
	**Fe**	-	-	1.00 0	−0.0008 0.995	0.004 0.973	0.047 0.686
	**Mg**	-	-	-	1.00 0	−0.389 **0.0005**	0.814 **<0.0001**
	**P**	-	-	-	-	1.00 0	−0.462 **<0.0001**
	**Zn**	-	-	-	-	-	1.00 0

^1^ The top number indicates the coefficient (*r*). ^2^ The bottom number indicates the significance (*p*-value).

## Data Availability

The data presented in this study are available from the corresponding author to protect producer privacy upon request.

## Abbreviations

As	Arsenic
Ba	Barium
Ca	Calcium
Cd	Cadmium
Co	Cobalt
Cr	Chromium
Cu	Copper
Fe	Iron
Mg	Magnesium
Mn	Manganese
Mo	Molybdenum
P	Phosphorus
SD	Standard Deviation
Se	Selenium
TM	Trace Mineral
Zn	Zinc
